# *Lasiodiplodia fici* sp. nov., Causing Leaf Spot on *Ficus altissima* in China

**DOI:** 10.3390/pathogens11080840

**Published:** 2022-07-27

**Authors:** GuiYan Xia, Ishara S. Manawasinghe, Alan J. L. Phillips, ChunPing You, Ruvishika S. Jayawardena, Mei Luo, Kevin D. Hyde

**Affiliations:** 1 Innovative Institute for Plant Health, Zhongkai University of Agriculture and Engineering, Guangzhou 510225, China; gyxia949@gmail.com (G.X.); ishara9017@gmail.com (I.S.M.); cpyou@zhku.edu.cn (C.Y.); 2 Center of Excellence in Fungal Research, Mae Fah Luang University, Chiang Rai 57100, Thailand; ruvi.jaya@yahoo.com; 3 School of Science, Mae Fah Luang University, Chiang Rai 57100, Thailand; 4 Faculdade de Ciências, Biosystems and Integrative Sciences Institute (BioISI), Universidade de Lisboa, Campo Grande, 1749-016 Lisbon, Portugal; alan.jl.phillips@gmail.com; 5 Key Laboratory of Fruit and Vegetable Green Prevention and Control in South-China, Ministry of Agriculture and Rural Affairs, Guangzhou 510225, China

**Keywords:** one new species, banyan trees, *Botryosphaeriaceae*, pathogenicity, tropical forest plants

## Abstract

High temperatures and the seasonality in tropical ecosystems favours plant pathogens, which result in many fungal diseases. Among these, diseases caused by *Botryosphaeriaceae* species are prominent as dieback, canker and leaf spots. In this research, we isolated one leaf-spot-causing *Botryosphaeriaceae* species from *Ficus altissima* leaves, which were collected in Guangzhou, Guangdong Province, China. Isolation and identification of the pathogen were based on morphological and molecular aspects. Based on multigene phylogenetic analysis of combined internal transcribed spacer (ITS), translation elongation factor 1-α gene (*tef1*) and beta-tubulin gene (*tub2*), the fungus associated with leaf spots on *F. altissima* is described as *Lasiodiplodia fici*, a novel species. Pathogenicity assays were conducted by inoculating the fungus onto detached shoots and plants under controlled environmental conditions. The results revealed that the *L. fici* isolates can infect the plant tissues under stress conditions by developing disease symptoms on detached shoots within three days. However, when it was inoculated onto the leaves of the host and grown in natural conditions, the progression of the disease was slow. The putative pathogen was re-isolated, and Koch’s assumptions were satisfied. This is the first report of *Lasiodiplodia* species causing disease on *Ficus altissima*. Results from the present study will provide additional knowledge on fungal pathogens associated with forest and ornamental plant species.

## 1. Introduction

Fungi are one of the key organisms in forest ecosystems. They live in the ecosystems as decomposers, mutualists and pathogens while contributing to continuous nutrient recycling and affecting biodiversity [1]. However, fungal pathogens associated with forest plants are understudied [2]. Among a wide range of pathogens, opportunistic pathogens are of great concern as they act when the environmental conditions are favourable for disease development [3,4,5]. *Botryosphaeriaceae* species are well-known opportunistic fungal pathogens causing devastating forest diebacks for a wide range of hosts [6,7]. Diseases caused by *Botryosphaeriaceae* species have higher significance as most of them infect the perennial parts of the plants [7]. Sydow and Theissen [8] introduced *Botryosphaeriaceae* in 1918, and the taxonomic status of *Botryosphaeriaceae* was controversial until it was reintegrated by Schoch et al. in 2006 [9]. However, following both morpho molecular aspects, *Botryosphaeriaceae* consists of 23 genera and over 100 species that are confirmed with DNA sequence data [7,10,11,12,13,14]. 

In 1894, Ellis introduced the *Lasiodiplodia* [15] with *L*. *tubericola* as the type species. Index Fungorum (March 2022) lists 84 *Lasiodiplodia* epithets. However, there are 71 species accepted with molecular data [7]. *Lasiodiplodia* is a cosmopolitan genus which has an extensive host and geographical range. Species of *Lasiodiplodia* are pathogenic on economically important fruit crops [6,16]. The pathogenicity of these species is varied, even within the same host in different countries. Even within a country, a single species can infect several different hosts [4]. As an example, *Lasiodiplodia theobromae* is one of the major pathogens of grapevine dieback, and the pathogenicity of this species varies in different countries [17,18,19,20,21]. In addition, *Lasiodiplodia* species have already been reported to cause disease in a broad range of forest plants, including *Acacia* sp. [12], *Eucalyptus* [22] and *Ficus* [23].

**Figure 1:**
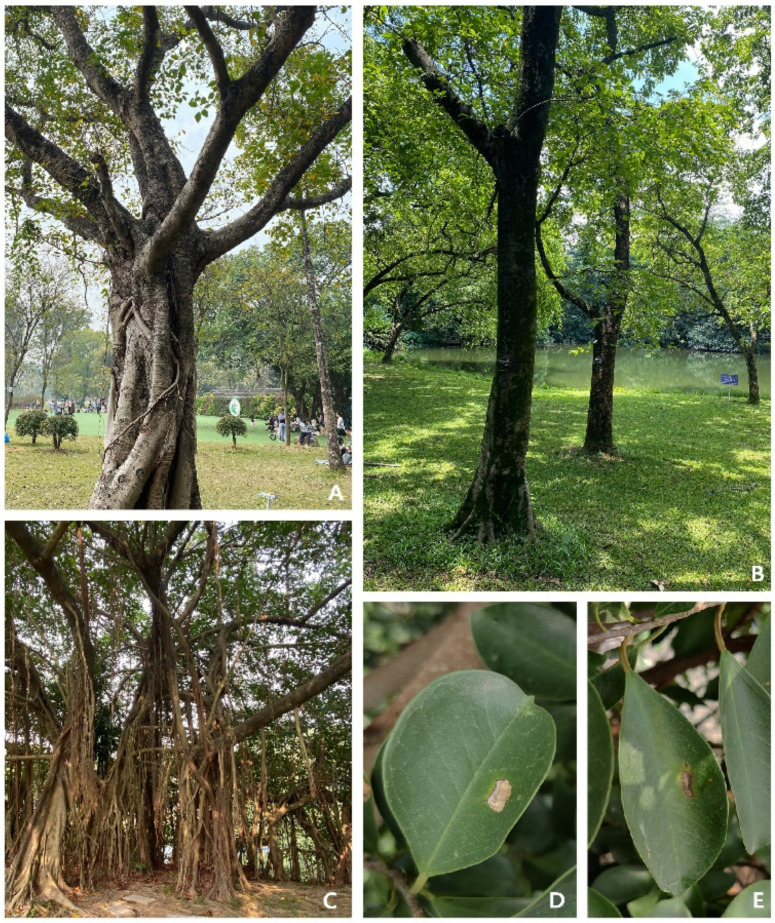
(**A**–**C**) Ornamental banyan trees are grown in South China Botanical Garden, Guangzhou, Guangdong, China. (**D**,**E**) Leafspot symptoms in the field.

The banyan tree (*Ficus*) is one of the genera of *Moraceae* and the only genus of the banyan Trib. *Ficeae* Trec [24]. This species is distributed from the southwest to the east and the south of China, while it is relatively rare in the rest of the country. This tree is a large tree reaching a height of 15–25 m; the bark is dark grey, the chest is about 50 cm in height with a wide crown and old trees often have rusty brown aerial roots [25]. The leaves are thin, tough, elongated and oval, with dark green surfaces and shiny and complete leaf arrangements [25]. The following are common characteristics of banyan trees: they come in pairs or are born with the axils of deciduous branches, they are yellow or reddish when mature, they are oblate, they have three basal bracts, they are broadly ovoid and they are persistent [25,26]. This plant is an important tropical forest tree as well as an ornamental plant (Figure 1). They are widely used as bonsai and landscaping trees [26]. Therefore, the isolation and characterisation of the diseases associated with this plant have both economic and ecological significance in China.

Banyan trees are highly susceptible to diseases and insect pests [27]. African snails, *Ficus* thrips, banyan grey silk moth, vermillion hair spot moth and banyan tree louse are the pests most widely associated with this host. Banyan trees are susceptible to several fungal diseases, including Anthracnose (*Colletotrichum* *gloeosporiodes*), a leaf spot caused by *Cercospora* spp. and *Corynespora* *cassiicola*, grey mould (*Botrytis* spp.), Phomopsis dieback (*Diplodia* spp.), Southern blight (*Sclerotium* *rolfsii*) and Verticillium wilt (*Verticilliumalboatrum)* [28,29].

In a sampling survey of banyan trees in Guangzhou City, Guangdong Province, China, a new leaf disease was observed. The objectives of this study were to (i) isolate and characterise the pathogen and (ii) confirmed the pathogenicity of isolated species. As result, a novel *Lasiodiplodia* species was identified by morphology and phylogenetic analysis based on *ITS*, *tef1* and *tub2.* Pathogenicity assays were conducted to confirm the pathogenicity, and Koch’s postulates were fulfilled. A detailed description and graphical illustration of new *Lasiodiplodia* species are provided.

## 2. Results

### 2.1. Sampling and Isolation 

During our tropical fungal survey at the South China Botanical Garden in Guangzhou City, Guangdong Province, China, a leaf spot disease was observed in banyan trees. A part of the infected leaf becomes brown and shows chlorosis. Then, the leaf spot becomes wider, and the plant tissue dies. When a leaf is severely damaged, leaf tissue becomes a grey/white transparent film (Figure 1). In a total of 20 banyan trees, 17 were reported to have these symptoms. From these infected trees, 10 plants were randomly selected, and from each plant, 10 leaves were collected. Almost all the leaves had similar disease symptoms (Figure 1D,E); thus, the samples were pooled, and 10 leaves were selected for tissue isolation. Tissue isolation resulted in 15 isolates, which were identical and resembled the morphology of *Lasiodiplodia* species. Thus, three strains (ZHKUCC 21-0125, ZHKUCC 21-0126 and ZHKUCC 21-0127) were selected for further analyses.

### 2.2. Morphological and Molecular Characterisation

**Figure 2:**
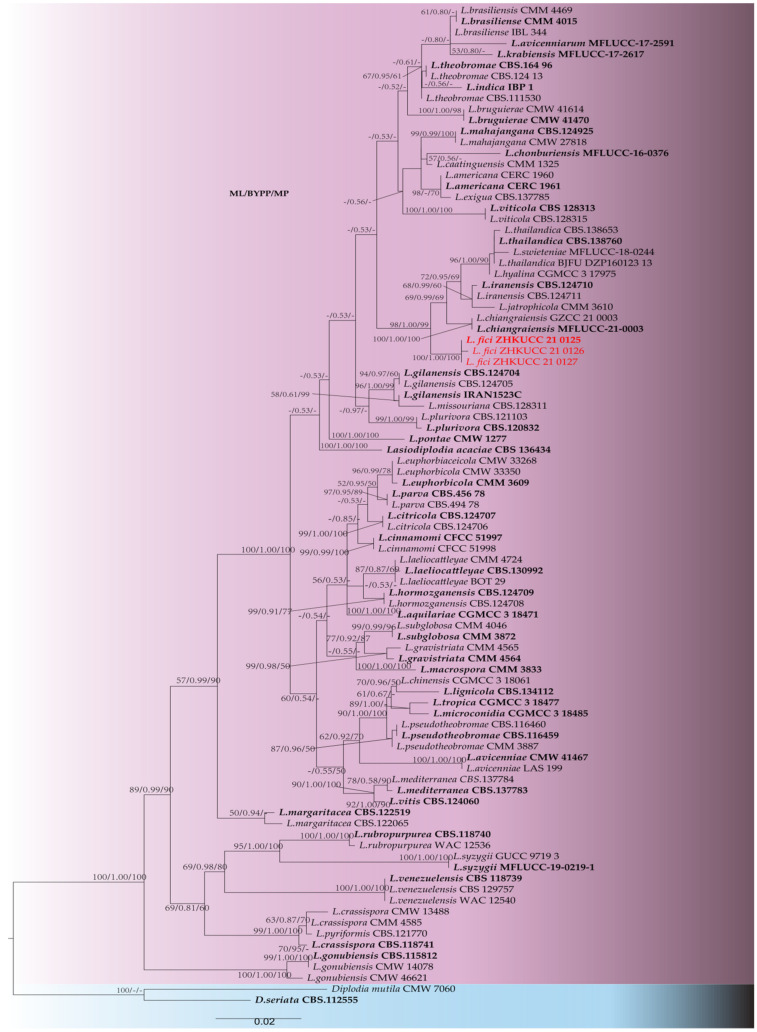
The best-scoring ML tree obtained from a heuristic search of the combined ITS, *tef1* and *tub2* sequence alignment of the *Lasiodiplodia* species. Bootstrap support values equal to or greater than 50% in MP, and ML and BYPP equal to or greater than 0.90 are shown as ML/BYPP/MP above the respective node. *Diplodia mutila* (CMW 7060) and *Diplodia seriata* (CBS 112555) are used as outgroup taxa. Ex-type strains are in bold and isolates belonging to this study are in red. Ex-type strains are bold.

Phylogenetic analyses were conducted using combined ITS, *tef1* and *tub2* sequence alignment of 91 *Lasiodiplodia* strains (including type strains), with *D*. *mutila* (CMW 7060) and *Diplodia seriata* (CBS 112555) as outgroup taxa. The resulting maximum likelihood (ML) tree shared a similar topology with maximum parsimony (MP) and Bayesian analysis (BYPP). The best-scoring ML tree is shown in Figure 2. The final dataset consists of 854 characters, of which 1139 characters are constants and 44 are parsimony informative. The maximal parsimonious analysis of the remaining 241 parsimonious information characters resulted in the 1000 most parsimonious trees of length 1001 steps (CI = 0.595, RI = 0.916, RC = 0.545, HI = 0.405). The best-scoring ML tree had the final likelihood value of -5342.638988. The matrix consisted of 357 distinct alignment patterns, with 7.89% of undetermined characters or gaps. Estimated base frequencies were as follows: A = 0.207301, C = 0.303614, G = 0.256996, T = 0.232089; substitution rates AC = 1.100912, AG =4.148566, AT = 1.475433, CG = 1.038445, CT = 5.970500, GT=1.000000; and gamma distribution shape parameter a = 0.195691. In the phylogenetic analysis, three isolates (ZHKUCC 21-0125, ZHKUCC 21-0126 and ZHKUCC 21-0127) from this study developed an independent clade from other known species. To confirm the novelty of isolated taxon, a strict morphological comparison was conducted, and sequence data were compared. We identified these isolates as novel *Lasiodiplodia* species. Species descriptions and illustrations are given below.

*Lasiodiplodia fici* G.Y. Xia, Manawas., M. Luo and K.D. Hyde *sp. nov.*

**Figure 3:**
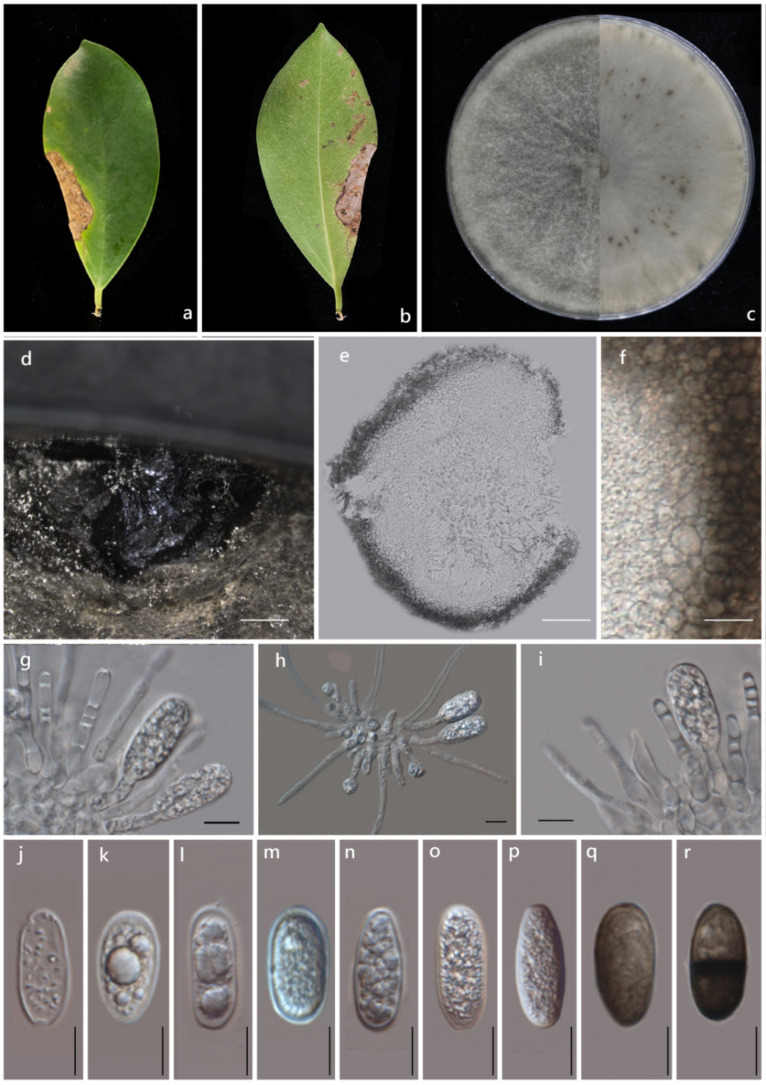
*Lasiodiplodia fici* (ZHKU 21-0092; Holotype). (**a**) Upper view of an infected leaf. (**b**) Reverse view of an infected leave. (**c**) Upper and reverse view of colonies on PDA after seven days. (**d**) A pycnidium on PDA after 28 days. (**e**) Vertical section through a pycnidium. (**f**) Pycnidial wall. (**g**–**i**) Conidiogenous cells with developing conidia. (**j**–**r**) Conidia. Scale bars: (**d**) = 1 mm, (**e**) = 500 um, (**f**) = 15 um, (**g**–**r**) = 10 um.

Index Fungorum number: IF 555265, Facesoffungi number: FoF 10815, (Figure 3).

**Holotype:** ZHKU 21-0092 

**Etymology:** Epithet refers to the host genus from which the fungus was isolated.

Pathogenic on *Ficus altissima* L. leaves. Sexual morph: Not observed. Asexual morph: *Conidiomata* pycnidial, produced on PDA within 4–8 wk, dark brown–black, unilocular, up to 3100 μm diam, immersed in the needle tissue, globose–subglobose, ostiolate, wall composed of several layers of dark brown *textura angularis*. *Peridium* 20–50 µm, composed of thick-walled, brown-black cells of *textura angularis*, thin inner wall. Cylindrical, septate, unbranched, ends rounded, formed among *conidiogenous* cells. *Conidiophores* absent. *Conidiogenous cells* 20–30 × 10–15 µm ($\bar{x}$ = 27 × 12 µm, n = 20), holoblastic, hyaline, smooth, thin-walled, cylindrical. *Conidia* 15–30 µm × 10–12 µm ($\bar{x}$ = 22 × 11 µm, n = 50), initially hyaline, aseptate, ellipsoid–ovoid, thin-walled with granular content, rounded at apex, base round or truncate, becoming dark brown, 1 septate with longitudinal striations.

**Culture characteristics:** Colonies on PDA reached 7 cm diameter after five days at 28 °C. The upper view was filamentous, flat and turbid. The aerial hyphae were fluffy, dense and turned grey and black with time. The back became black.

**Materials examined:** China, Guangdong Province, Guangzhou City, South China Botanical Garden on the dead leaves of *Ficus altissima* L. *(Moraceae*), May 12, 2021, X. Guiyan, (ZHKU 21-0092 Holotype), living cultures ZHKUCC 21-0125 ex-type; ZHKUCC 21-0126, ZHKUCC 21-0127.

Note: Three isolates obtained in this study were phylogenetically closely related to *L. iranensis*, *L. chiangraiensis* and *L. thailandica*. However, isolates from this study formed a distinct linage with 100% ML, 100% MP and 1.0 BYPP support (Figure 2). Morphologically, these species can be distinguished from the dimensions of their conidia (Table 1), in which *Lasiodiplodia fici* developed conidia with a higher L/W ratio. In terms of the nucleotide comparison, *Lasiodiplodia fici* (ZHKUCC 21-0125) and *Lasiodiplodia chiangraiensis* (MFLUCC 21-0003) differed in one base pair (bp) in ITS, seven in *tef1* and two in *tub2*. *Lasiodiplodia fici* (ZHKUCC 21-0125) and *Lasiodiplodia iranensis* (CBS 12471) differed in one base pair (bp) in ITS, 13 in *tef1-*α, and CBS 124710 did not have *tub2* sequence. *Lasiodiplodia fici* (ZHKUCC 21-0125) and *Lasiodiplodia thailandic* (CPC 2279) differed in one base pair (bp) in ITS and fifteen in *tef1*, but *tub2* of these two species are identical. Based on these polyphasic approaches, we introduced *Lasiodiplodia fici* as a new species. 

### 2.3. Pathogenicity Assay 

**Figure 4:**
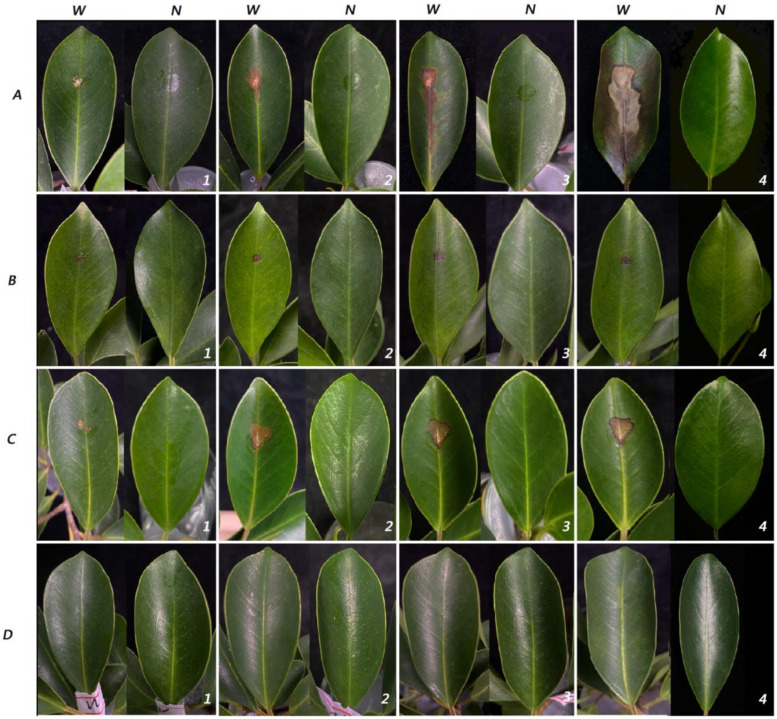
Pathogenicity test results of *Lasiodiplodia* *fici* inoculated to detached shoots. (**A**) ZHKUCC 21-0125; (**B**) ZHKUCC 21-0126; (**C**) ZHKUCC 21-0127; (**D**) control. (**1**) One day after inoculation (dpi); (**2**) 3 dpi; (**3**) 5 dpi; (**4**) 7 dpi. **W**—wounded; **N**—non-wounded.

**Figure 5:**
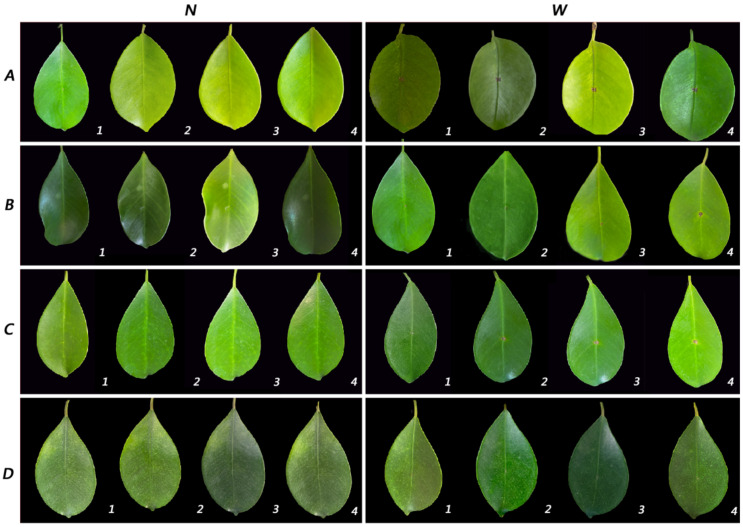
Pathogenicity test results of *Lasiodiplodia* *fici* inoculated to host plant. (**A**) ZHKUCC 21-0125; (**B**) ZHKUCC 21-0126; (**C**) ZHKUCC 21-0127; (**D**); control. (**1**) One day after inoculation (dpi); (**2**) 3 dpi; (**3**) 5 dpi; (**4**) 7 dpi. **W**—wounded; **N**—non-wounded.

In this study, sporulation of *Lasiodiplodia fici* was difficult to perform, even after trying different media (MEA, WA and PDA) and pine needles. Therefore, the pathogenicity test was carried out by the mycelial plug method. From actively growing cultures on PDA, mycelial plugs 50 mm in diameter and 20 mm in height were used. After 24 h, all wounded inoculated detached shoots had small lesions. Three days later, leaves fell from one infected shoot of the isolate ZHKUCC 21-0127. Then, leaf spots enlarged. Five days later, all infected leaves of three isolates had fallen off, while non-inoculated leaves did not. The disease development and progress of the isolate ZHKUCC 21-0125 were higher than the other two strains. No disease symptoms developed on any of the controls. All non-wounded samples were the same as the control leaves without any disease. Under field conditions, wounded leaves inoculated with mycelial and developed the disease slowly. After seven days of inoculation, ZHKUCC 21-0125 produced the largest lesion (Figure 4). However, disease establishment and progress were slower on plants inoculated in their natural habitat. All diseased leaf tissues were subjected to re-isolation of the pathogen, and the species was confirmed as *Lasiodiplodia fici* using sequence data (Figure 5).

## 3. Discussion

In this paper, a new *Lasiodiplodia* species is described from *Ficus altissima* in China. The fungus was shown to be pathogenic, causing leaf spots similar to the ones from which it was isolated. Correct species identification is important in plant pathology for early detection and development of management strategies [33]. Species delineation in *Botryosphaeriaceae* genera, such as *Lasiodiplodia* has been the subject of debate in recent years. Morphology and molecular phylogeny have become important criteria in this genus to resolve species [6]. In the phylogenetic analysis of this study, *L. fici* clusters together with *Lasiodiplodi iranensis*, *L. chiangraiensis* and *L. thailandica*. Together, these four species develop a distinct clade from other *Lasiodiplodia* species in the tree. Even though, within this cluster, these species developed independent lineages, their morphology and sequence data are very similar (Table 1). This clustering variation might be a result of the host specificity of species. *Lasiodiplodia iranensis* was introduced from *Salvadora persica* from Iran, and both *L. chiangraiensis* and *L. thailandica* were recorded from Thailand, while *L. thailandica* is from *Mangifera indica*, while *L. chiangraiensis* from dead wood. The new species from this study was found on *Ficus altissima* in China. Further studies are required to define the limits of species in *Lasiodiplodia* and to determine whether the four species in this clade are distinct or if they constitute variation within a single species.

Up until today, four *Lasiodiplodia* species have been recorded on *Ficus*: *L. mahajangana* on *Ficus opposite* [34], *L. pseudotheobromae* on *Ficus racemosa* [32], *L. theobromae* on *Ficus benghalensis* [35], *Ficus carica* [36,37,38,39], *Ficus elastica* [40], *Ficus insipida* [41], *Ficus lyrata* [42], *Ficus nitida* [35], *Ficus opposita* [43], *Ficus sp.* [37] and *L. venezuelensis* on *Ficus insipida* [41]. Moreover, 19 *Botryosphaeriaceae* species have been reported on Banyan trees [33]. These species are *Botryosphaeria dothidea*, *B. fabicerciana*, *Diplodia seriata*, *Dothiorella alpina*, *Do. citrimurcotticola*, *Do. plurivora*, *Lasiodiplodia citricola*, *L. guilinensis*, *L. huangyanensis*, *L. iraniensis*, *L. linhaiensis*, *L. microconidia*, *L. ponkanicola*, *L. pseudotheobromae*, *L. theobromae*, *Neodeightonia subglobosa*, *Neofusicoccum parvum* and *Sphaeropsis linhaiensis* [29]. The new species described here will be an addition to this.

In this study, we observed that *L. fici* becomes more aggressive and forms lesions within three days when mycelial plugs were inoculated onto the detached shoots. However, when it was inoculated on the host in its natural habitat, disease progression was slower. Thus, our results confirm the opportunistic nature of this fungal group. Isolate ZHKUCC 21-0125 was more aggressive than the other two strains, showing the variations in pathogenicity among the three strains used in this study. This suggests that *Lasiodiplodia* might have inter-species variation based on pathogenicity as well. Previous studies have shown that several other *Botryosphaeriaceae*, such as *Botryosphaeria* [44] and *Neofusicoccum* [45], as well as *Lasiodiplodia* [46], have high genetic diversity. However, in this study, we used the mycelial plug method, and the use of the mycelial plug method over spore suspension also leads to false-positive conclusions, in which weak pathogens can be more aggressive when supplied with a large nutrient base in the agar growth medium [4]. 

In the present study, one of the main problems we faced was the difficulty to sporulate this fungus. We tried different media (PDA, MEA, WA), adding pine needles to the medium as well as adding host tissues to sporulate this fungus. However, it took over three months for out observations of the fruiting bodies to confirm the morphology. This is the main reason why we did not perform pathogenicity using spore suspension, as we could not obtain spores to make 1 × 10^6^ spore suspension. In addition to that, we did not observe any type of fruiting bodies on the leaf surface in this study. Up until today, it is debatable how they become pathogenic and what their infection mechanisms are. *Lasiodiplodia* species do not produce appressoria or any other structures to become pathogenic on the host. Thus, most studies related to *Lasiodiplodia* and most of the Botryosphaeriaceae taxa showed similar observations as we did [3,4,16,20]. These species are opportunistic fungal pathogens; when the host becomes stressed due to environmental conditions, they are supposed to develop the disease. Therefore, it is necessary to have natural connections to the host tissues, such as stromata or physical injury. This is the main reason for our observations in the pathogenicity essay.

China has a rich and diverse vegetation cover; thus, identifying different fungal species associated with plants is of great significance for the ecological control of plants. *Moraceae* represents many ecological and economically important plants, including *Ficus* spp. and Mulberries [47]. However, there is a limited number of studies on microfungi associated with these hosts, especially with forest plants such as *Ficus*. Therefore, the new species described here will provide new insight into the disease occurrence in forest plants as well as *Moraceae* hosts. The new species record of *Lasiodiplodia* in *Botryosphaeriaceae* can enrich domestic resources and provide a certain database which is of great significance for enriching fungal species in China. 

## 4. Materials and Methods

### 4.1. Samples Collection and Isolation

Samples were collected from the South China Botanical Garden in Guangzhou City, Guangdong Province (E 113°21′56″ N 23°11′24″). The sample was brought into the laboratory, and the pathogen was isolated using tissue dissociation [33,48,49]. The infected tissue was thoroughly cleaned with sterile water. Then, the samples were cut with sterilized scissors into small pieces (5 mm^2^) that included both infected and healthy tissues. After that, the leaf cuttings were washed with 75% ethanol for 30 s, 10% NaOCl for 30 s and followed by three times with sterilised water. After that, cuttings were dried on sterilized filter paper, transferred to a PDA plate and incubated for 5–7 days at 25 °C. When the mycelia appeared in the tissue samples, the purification was carried out by hyphal tip isolation. Purification was preformed 3–5 times to obtain pure cultures. All pure cultures obtained in this study and Herbarium materials (as dry cultures) were deposited in the Culture Collection and the Herbarium of Zhongkai University of Agricultural Engineering (ZHKUCC), Guangzhou City, Guangdong, China.

### 4.2. DNA Extraction, Amplification and Sequencing

Total genomic DNA was extracted from the sterile 7-day-old pure cultures using MagPure Plant AS Kit (Aidlab Biotechnologies Co., Ltd., Beijing, China) following the manufacturer’s protocols. Three gene regions, ITS, *tef1* and *tub2*, were used for PCR amplification of the isolated strains. The PCR mixtures consisted of 1 μL genomic DNA, 1× NH4 reaction buffer Guangzhou Meiji Biotechnology Co., Ltd., Guangzhou, China), 2 mM MgCl2, 40 μM of each dNTP, 0.2 μM of each primer and 0.5 U Taq DNA polymerase (Guangzhou Meiji Biotechnology Co., Ltd., China), in a total volume of 12.5 μL. Each gene region ITS, *tef1* and *tub2* was amplified and sequenced following White et al. [12], Carbone et al. [50] and Glass et al. [51], respectively. The PCR was performed in a C1000 TouchTM thermal cycler (Guangzhou Hongtu instrument Co., Ltd., Guangzhou, China), and the positive implications were observed after 1% agarose gel electrophoresis under ultraviolet light using GelDoc XR^+^ (BIO-RAD, Hercules, CA, USA). All positive amplicons were sequenced by Tianyi (Guangzhou, China) Co., Ltd. Sequence data generated in this study were deposited in NCBI Genbank (Supplementary Table S1).

### 4.3. Phylogenetic Analysis

BioEdit v.7.0.5.2 [52] was used to check the sequence quality by checking sequence chromatograms. Then, the sequences were combined with BioEdit v.7.0.5.2. Initial species confirmation was performed using the National Centre for Biotechnology Information (NCBI) search engine GenBank BLASTn (https://blast.ncbi.nlm.nih.gov/Blast.cgi (accessed on 18 March 2022)). Relevant sequences of species belonging to *Lasiodiplodia* and outgroup taxa were downloaded from NCBI following Xiao [53]. Using MAFFT v. 7 webserver (http://mafft.cbrc.jp/alignment/server (accessed on 20 March 2022), sequences from this study aligned together with the downloaded sequences. Then, files were combined in ITS, *tef1* and *tub2* order by BioEdit v.7.0.5.2 [52]. In the present study, maximum likelihood (ML), maximum parsimony (MP) and Bayesian analysis were used in phylogenetic analyses.

Maximum parsimony analysis was performed using PAUP (phylogenetic analysis using parsimony) v.4.0b10 [54]. Here, the heuristic search was performed with tree bisection–reconnection (TBR) branch swapping and 1000 random sequence additions. In the alignment, ambiguous regions were excluded. Then, gaps were treated as missing data. Tree stability was evaluated by 1000 bootstrap replications. The most parsimonious trees were saved after branches of zero length collapsed. Tree length (TL), consistency index (CI), retention index (RI), relative consistency index (RC) and homoplasy index (HI) were calculated.

The best model of evolution was determined by MrModeltest v. 2.2 for each gene. Maximum likelihood analyses via RAxML v. 8.2.12 [55] were accomplished using RAxML-HPC2 on XSEDE v. 8.2.8 [55,56] using the CIPRES Science Gateway platform [57]. GTR + I + G evolution model was used with 1000 non-parametric bootstrapping iterations. MrBayes v.3.0b4 was [55] used for the Bayesian analyses. The Markov Chain Monte Carlo sampling (BMCMC) analysis was conducted with four simultaneous Markov chains. The MCMC heated chain was set with a “temperature” value and run for 1,000,000 generations. Trees were sampled at every 100th generation. From the 10,000 trees obtained, the first 2000, representing the burn-in phase, were discarded. To calculate posterior probabilities, the remaining 8000 trees were used. New data were submitted to the Faces of Fungi database [58] and Index Fungorum (http://www.indexfungorum.org (accessed on 20 March 2022). New species were described following Jayawardena et al. [59] and Manawasinghe et al. [60].

### 4.4. Pathogenicity Assay

The pathogenicity of the three isolates obtained in this study (ZHKUCC 21-0125, ZKUCC 21-0126 and ZKUCC 21-0127) was tested on healthy *Ficus* leaves. The inoculum was mycelial plugs. We did not use conidial suspension inoculation since our isolates did not produce enough conidia to make a 1 × 10^6^ spore suspension. We used detached shoots and plants grown in natural habitats. Healthy leaves with the same developmental stages were selected. The leaves were surface sterilized with 75% alcohol and wounded by pricking with a sterilized needle. Another set of unwounded leaves was inoculated. Mycelial plugs 5 mm diameter were applied to both wounded and non-wounded leaves and covered with parafilm. Non-colonised PDA plugs were used in the control. There were three replicates for each isolate and control. Once the disease was developed, the pathogen was re-isolated to confirm Koch’s postulates.

## Figures and Tables

**Table 1:** A morphological comparison of conidial dimensions of *Lasiodiplodia fici* and its phylogenetically closely related species.

	Conidial Dimensions (μm)	L/W Ratio	Reference
*Lasiodiplodia fici*	17–28 × 9–14	2.0	This study
*Lasiodiplodia chiangraiensis*	22–27 × 13–15	1.9	[30]
*Lasiodiplodia iranensis*	17–23 × 11–14	1.6	[31]
*Lasiodiplodia thailandica*	22–25 × 13–15	1.7	[32]

## Data Availability

The sequence data generated in this study are deposited in NCBI GenBank (https://www.ncbi.nlm.nih.gov/genbank). All accession numbers are given in Supplementary Table S1.

## Supplementary Materials

The following supporting information can be downloaded at: https://www.mdpi.com/article/10.3390/pathogens11080840/s1, Table S1: GenBank accession numbers of the isolates included in the phylogenetic analysis.
