# Supplementary material for: Lasiodiplodia fici sp. nov., Causing Leaf Spot on Ficus altissima in China
Source: Pathogens. 2022 Jul 27;11(8):840. doi: 10.3390/pathogens11080840 (PMC9412989; doi:10.3390/pathogens11080840)
Supplement: Supplementary file 1 [file pathogens-11-00840-s001.zip › pathogens-1798841-supplementary.pdf]

Supplementary Table S1. GenBank accession numbers of the isolates included in the phylogenetic analysis.

Species	Isolate number	Host	GenBank accession number		
			ITS	*tef1*	*tub2*
Lasiodiplodia acaciae	**CBS 136434***	*Acacia* sp.	MT587421	MT592133	MT592613
L. aquilariae	CGMCC 3.18471*	*Aquilaria crassna*	KY783442	KY848600	N/A
L.americana	CERC 1961 = CFCC 50065 *	*Pistachia vera*	KP217059	KP217067	KP217075
L.americana	CERC 1960 = CFCC 50064	*Pistachia vera*	KP217058	KP217066	KP217074
L.avicenniae	CMW 41467 *	*Avocennia marina*	KP860835	KP860680	KP860758
L.avicenniae	LAS 199	*Avocennia marina*	KU587957	KU587947	KU587868
L. avicenniarum	MFLUCC 17-2591*	*Avicennia marina*	MK347777	MK340867	N/A
L.brasiliense	CMM 4015 *	*Mangifera indica*	JX464063	JX464049	N/A
L.brasiliense	IBL 344	*Adansonia madagascariensis*	KT151808	KT151802	KT151805
L. brasiliensis	CMM 4469	*Anacardium occidentale*	KT325574	KT325580	N/A
L.bruguierae	CMW 41470*	*Bruguiera gymnorrhiza*	KP860833	KP860678	KP860756
L.bruguierae	CMW 41614	*Bruguiera gymnorrhiza*	KP860834	KP860679	KP860757
L. caatinguensis	CMM 1325	*Citrus sinensis*	KT154760	KT008006	KT154767
L. chiangraiensis	MFLUCC 21-0003*	Unknown host	MW760854	MW815630	MW815628
L. chiangraiensis	GZCC 21-0003	Unknown host	MW760853	MW815629	MW815627
L. chinensis	CGMCC 3.18061	Woody branch	KX499889	KX499927	KX500002
L. chonburiensis	MFLUCC 16-0376*	*Pandanus* sp.	MH275066	MH412773	MH412742
L.cinnamomi	CFCC 51997 *	*Cinnamomum camphora*	MG866028	MH236799	MH236797
L.cinnamomi	CFCC 51998	*Cinnamomum camphora*	MG866029	MH236800	MH236798
L.citricola	CBS 124707 = IRAN 1522C *	*Citrus* sp.	GU945354	GU945340	KP872405
L.citricola	CBS124706 = IRAN 1521C	*Citrus* sp.	GU945353	GU945339	KP872406
L. crassispora	CMW 13488	*Eucalyptus urophylla*	DQ103552	DQ103559	KU887507
L.crassispora	CBS 118741 = WAC12533 *	*Santalum album*	DQ103550	EU673303	KU887506
L.crassispora	CMM 4585	–	MG954354	MG979520	MG979552
L. euphorbiaceicola	CMW 33268	*Adansonia*	KU887131	KU887008	KU887430
L.euphorbicola	CMM 3609 *	*Jatropha curcas*	KF234543	KF226689	KF254926
L.euphorbicola	CMW 33350	*Adansonia digitata*	KU887149	KU887026	KU887455
L. exigua	CBS 137785	*Quercus ilex*	KJ638317	KJ638336	KU887509
L. fici	ZHKUCC 21-0125*	*Ficus altissima*	ON178662	ON599008	ON599011
L. fici	ZHKUCC 21-0126	*Ficus altissima*	ON178664	ON599007	ON599010
L. fici	ZHKUCC 21-0127	*Ficus altissima*	ON178663	ON599006	ON599009
L. gilanensis	CBS 124704*	*Citrus sp.*	GU945351	GU945342	KU887511
L.gilanensis	CBS 124704 = IRAN1523C= UCCE 940B *	*Citrus* sp.	KX906851	KX906853	KX906849
L.gilanensis	CBS 124705 = IRAN 1501C	*Citrus* sp.	GU945352	GU945341	KP872412
L. gonubiensis	CMW 14078	*Syzygium cordatum*	AY639594	DQ103567	EU673126
L.gonubiensis	CBS 115812 = CMW 14077 *	*Syzygium cordatum*	AY639595	DQ103566	DQ458860

L.gonubiensis	CMW 46621 = MTU 56	*Syzygium cordatum*	KY052944	KY024623	KY000126
L.gravistriata	CMM 4564 *	*Anacardium humile*	KT250949	KT250950	N/A
L.gravistriata	CMM 4565	*Anacardium humile*	KT250947	KT266812	N/A
L.hormozganensis	CBS 124709 = IRAN 1500C *	*Olea* sp.	GU945355	GU945343	KP872413
L.hormozganensis	CBS 124708 = IRAN 1498C	*Mangifera indica*	GU945356	GU945344	KP872414
L. hyalina	CGMCC 3.17975	*Acacia confusa*	KX499879	KX499917	KX499992
L.indica	IBP 1 *	*Angiospermous tree*	KM376151	N/A	N/A
L.iranensis	CBS 124710 = IRAN 1520C *	*Salvadora persica*	GU945348	GU945336	KU887516
L.iranensis	CBS 124711 = IRAN 1502C = CMM 4603	*Juglans* sp.	GU945347	GU945335	MG979537
L. jatrophicola	CMM 3610	*Jatropha curcas*	KF234544	KF226690	KF254927
L. krabiensis	MFLUCC 17-2617*	*Bruguiera* sp.	MN047093	MN077070	N/A
L. laeliocattleyae	CBS 130992*	*Mangifera indica*	KU507487	KU507454	KU887508
L. laeliocattleyae	BOT 29	*Mangifera indica*	JN814401	JN814428	N/A
L. laeliocattleyae	CMM 4724	*Vitis vinifera*	MG954343	MG979508	MG979541
L.lignicola	CBS 134112= MFLUCC 11-0435 *	Dead wood	JX646797	KU887003	KT852958
L.macrospora	CMM 3833 *	*Jatropha curcas*	KF234557	KF226718	KF254941
L.mahajangana	CBS 124925 = CMW 27801 *	*Terminalia catappa*	FJ900595	FJ900641	FJ900630
L.mahajangana	CMW 27818	*Terminalia catappa*	FJ900596	FJ900642	FJ900631
L.margaritacea	CBS 122519 = CMW 26162 *	*Adansonia gibbosa*	EU144050	EU144065	KX464903
L.mediterranea	CBS 122065	*Adansonia gibbosa*	EU144051	EU144066	N/A
L.mediterranea	CBS 137783 *	*Holm oak*	KJ638312	KJ638331	KU887521
L.mediterranea	CBS137784	*Grapevine*	KJ638311	KJ638330	KU887522
L.microconidia	CGMCC 3.18485 *	*Aquilaria crassna*	KY783441	KY848614	N/A
L. missouriana	CBS 128311	*Vitis vinifera*	HQ288225	HQ288267	HQ288304
L. parva	CBS 456.78 *	Cassava-field soil	EF622083	EF622063	KU887523
L. parva	CBS 494.78	Cassava-field soil	EF622084	EF622064	EU673114
L.plurivora	CBS 120832 = STE-U5803 *	*Prunus salicina*	EF445362	EF445395	KP872421
L.plurivora	CBS 121103 = STE-U4583	*Vitis vinifera*	AY343482	EF445396	KP872422
L.pontae	CMW 1277 = IBL12 *	*Spondias purpurea*	KT151794	KT151791	KT151797
L. pseudotheobromae	CBS 116460	*Acacia mangium*	EF622078	EF622058	KU198428
L.pseudotheobromae	CBS 116459 *	*Gmelina arborea*	EF622077	EF622057	EU673111
L. pseudotheobromae	CMM 3887	*Jatropha curcas*	KF234559	KF226722	KF254943
L. pyriformis	CBS 121770	*Acacia mellifera*	EU101307	EU101352	KU887527
L.rubropurpurea	CBS 118740 = CMW 14700 = WAC 12535 *	*Eucalyptus grandis*	DQ103553	DQ103571	KU887529
L.rubropurpurea	WAC 12536 = CMW 15207	*Eucalyptus grandis*	DQ103554	DQ103572	KP872425
L.subglobosa	CMM 3872 *	*Jatropha curcas*	KF234558	KF226721	KF254942
L.subglobosa	CMM 4046	*Jatropha curcas*	KF234560	KF226723	KF254944
L. swieteniae	MFLUCC 18-0244	*Swietenia mahagoni*	MK347789	MK340870	MK412877
L. syzygii	GUCC 9719.1*	*Wax apple*	MT990531	MW016943	MW014331
L. syzygii	GUCC 9719.3	*Syzygium samarangense*	MW081992	MW087102	MW087105
L. thailandica	CBS 138760*	*Mangifera indica*	KJ193637	KJ193681	N/A

L. thailandica	CBS 138653	*Phyllanthus acidus*	KM006433	KM006464	N/A
L. thailandica	BJFU DZP160123-13	*Albizia chinensis*	KY676789	KY676798	KY751301
L.theobromae	CBS 164.96 *	Fruit along caral reef coast	AY640255	AY640258	KU887532
L.theobromae	CBS 111530	*Leucospermum* sp.	EF622074	EF622054	KU887531
L.theobromae	CBS 124.13	–	DQ458890	DQ458875	DQ458858
L. tropica	CGMCC 3.18477*	*Aquilaria crassna*	KY783454	KY848616	KY848540
L. venezuelensis	WAC 12540	*Acacia mangium*	DQ103548	DQ103569	KU887534
L.venezuelensis	CBS 118739 = CMW 13511 = WAC 12539 *	*Acacia mangium*	DQ103547	EU673305	KU887533
L.venezuelensis	CBS 129757	*Acacia mangium*	JX545102	JX545122	JX545142
L.viticola	CBS 128313 = UCD 2553AR *	*Vitis vinifera*	HQ288227	HQ288269	HQ288306
L.viticola	CBS 128315 = UCD 2604MO	*Vitis vinifera*	HQ288228	HQ288270	HQ288307
L. vitis	CBS 124060*	*Vitis vinifera*	KX464148	KX464642	KX464917
Diplodia mutila	CMW 7060	*Fraxinus excelsior*	AY236955	AY236904	AY236933
D. seriata	CBS 112555*	*Vitis vinifera*	AY259094	AY573220	DQ458856

* Indicates ex-type/ex-epitype isolates. The new species is indicated in bold. Abbreviations of isolates and culture collections: BOT—Personal number of S. Denman; CBS—Centraalbureau voor Schimmelcultures, Utrecht, Netherlands; CFCC—China Forestry Culture Collection Center, Beijing, China; CGMCC—China General Microbiological Culture Collection Center; CMM—Culture Collection of Phytopathogenic Fungi "Prof. Maria Menezes", Universidade Federal Rural de Pernambuco, Recife, Brazil; CMW—Culture collection of the Forestry and Agricultural Biotechnology Institute (FABI) of the University of Pretoria, Pretoria South Africa; GZCC—Guizhou Culture Collection, Guiyang, China; MFLUCC—Mae Fah Luang University Culture Collection, Chiang Rai, Thailand; STE-U—Culture Collection of the Department of Plant Pathology, University of Stellenbosch, South Africa; UCD—University of California, Davis, Plant Pathology Department Culture Collection; WAC—Department of Agriculture Western Australia Plant-Pathogen Collection, Perth, Australia. Sequences produced in this study are shown in red. * ex-type or ex-epitype culture.
